# An Asset for Food Safety: The Knowledge Behind the Physiological Alterations Induced by ETEC Enterotoxins

**DOI:** 10.3390/foods14213651

**Published:** 2025-10-26

**Authors:** Maria Margarida Barros, Ana Maria Campos, Joana Castro, Ricardo Oliveira, Daniela Araújo, Divanildo Outor-Monteiro, Carina Almeida

**Affiliations:** 1 National Institute for Agrarian and Veterinary Research (INIAV), Rua dos Lagidos, 4485-655 Vila do Conde, Portugal; margarida.barros@iniav.pt (M.M.B.); anamaria.campos@iniav.pt (A.M.C.); joana.castro@iniav.pt (J.C.); ricardo.oliveira@iniav.pt (R.O.); daniela.araujo@iniav.pt (D.A.); 2 Veterinary and Animal Research Centre (CECAV), University of Trás-os-Montes and Alto Douro, 5000-801 Vila Real, Portugal; divanildo@utad.pt; 3 Centre of Biological Engineering, University of Minho, 4710-057 Braga, Portugal; 4 Laboratory for Process Engineering, Environment, Biotechnology and Energy (LEPABE), Associate Laboratory in Chemical Engineering (ALiCE), Faculty of Engineering, University of Porto, Rua Dr. Roberto Frias, 4200-465 Porto, Portugal

**Keywords:** ETEC enterotoxins, physiological alterations, food safety

## Abstract

Foodborne pathogens represent a significant public health risk in both developed and developing countries. Among these pathogens, enterotoxigenic *Escherichia coli* (ETEC) is a major cause of diarrhea in humans and one of the leading causes of mortality in newly weaned pigs. The main sources of ETEC contamination include environments with poor hygiene and contaminated water, meat, cereals, and vegetables. Therefore, this review manuscript focuses on the pathogenesis of ETEC in humans and pigs. The main virulence factors responsible for ETEC-associated infections, such as colonization factors and toxins, will be described for both species, with particular emphasis on the toxins as well as, their classification and structural characterization. More specifically, this study will outline the main physiological alterations and adaptive mechanisms induced by these enterotoxins, namely heat-stable toxin (ST) and heat-labile toxin (LT), in the three most affected systems: the gastrointestinal system, the enteric nervous system (ENS), and the immune system. This set of findings provides a deeper insight into the pathogenesis of this relevant foodborne pathogen, which is crucial for empowering food scientists and stakeholders to more effectively mitigate associated risks. As such, it provides valuable understanding of toxin activity, serving as a means to raise awareness of food safety practices and strengthening risk communication, surveillance and intervention strategies, thereby ensuring consumer protection. Additionally, this knowledge enables the development of preventive strategies to reduce ETEC infections, thereby decreasing the need for clinical management among consumers exposed to this bacterium. Ultimately, it contributes to the preservation of public health, the reduction of antimicrobial use, and the lowering of antimicrobial resistance gene prevalence.

## 1. Introduction

*Escherichia coli* is typically a harmless commensal microorganism; however, through the acquisition of diverse genetic elements, it can evolve into a highly adapted and virulent pathogen. Pathogenic *E. coli* can lead to a range of diseases in the host, ranging from gastroenteritis to extraintestinal infections affecting the urinary tract, bloodstream, and central nervous system [1]. These diseases may affect either animals or humans worldwide and comprise two main classes of pathovars: diarrheagenic *E. coli* and extraintestinal *E. coli* [2]. The following six pathovars are classified as diarrheagenic: enteropathogenic *E. coli* (EPEC), enterohaemorrhagic *E. coli* (EHEC), enterotoxigenic *E. coli* (ETEC), enteroinvasive *E. coli* (EIEC), enteroaggregative *E. coli* (EAEC), and diffusely adherent *E. coli* (DAEC). Of particular importance is the Shiga toxin–producing *E. coli* (STEC) pathotype, which largely overlaps with EHEC. Cattle serve as its major reservoir and represent the primary source of human foodborne outbreaks associated with this pathogen [3]. While uropathogenic *E. coli* (UPEC) and neonatal meningitis *E. coli* (NMEC) are the two most common pathovars within the extraintestinal *E. coli*-associated infections [1], it is important to mention that other pathovars have been recognized, but their mechanisms of pathogenicity are not well established. Additionally, several virulence strategies are shared among distinct *E. coli* pathovars, such as adhesion to host cells achieved through fimbriae or pili (with the exception of the EIEC pathovar), followed by the disruption of host cell processes. Although each pathovar has its typical processes of attaching to and exploiting host cells [1], the present study focuses on ETEC, highlighting its virulence mechanisms and the physiological alterations induced by the enterotoxins produced in humans and pigs.

Specifically, ETEC strains affecting humans are a major cause of childhood diarrhea and represent the leading cause of travel-associated diarrhea in developing countries, especially in areas with inadequate food and water hygiene [2,4]. According to annual estimates, ETEC is responsible for around 220 million episodes of diarrhea globally, with approximately 75 million episodes occurring in children under five years of age. This burden results in between 18,700 and 42,000 deaths in children younger than five, according to estimates from the Institute for Health Metrics and Evaluation (IHME) and Maternal Child Epidemiology (MCEE), respectively [5].

ETEC also has a major impact on livestock, particularly swine, which are among the animal species most affected. In pigs, these strains are responsible for neonatal and postweaning diarrhea, and represent one of the most significant threats to the swine industry, causing significant economic losses associated with morbidity, mortality, decreased weight gain, the rising cost of treatments, vaccinations, and feed supplementation [6,7,8,9].

In both humans and swine, pathogenesis is initiated with ETEC ingestion via the oral route and then passes through the digestive system until it reaches the small intestine. In this intestinal section, colonization is initiated by attaching fimbria adhesins to specific receptors present on the epithelium or its mucosal coating. Once established, ETEC produces and releases enterotoxins inside the epithelial cells, known as enterocytes, which stimulates the secretion of water and electrolytes into the intestinal lumen, ultimately resulting in watery diarrhea and, in severe cases, death [10,11,12].

In humans, ETEC contamination is mitigated through hygienic food processing and handling, rigorous sanitation of facilities and equipment, and bactericidal treatments such as cooking or pasteurization. Consumer awareness has also become a key factor, with safer food choices and hygienic habits helping to prevent infection, both locally and while traveling [13,14,15,16]. It is therefore important to highlight that preventive strategies depend mainly on individual practices, including avoiding raw or undercooked food, ensuring safe drinking water, and maintaining good hygiene [17]. It is also relevant to mention that these strategies should also beapplied to prevent viral gastroenteritis, as noroviruses are the leading cause of non-bacterial human gastroenteritis in developed countries [18].

Similarly, in animal production systems, good agricultural practices are essential to prevent ETEC infection and propagation in pigs. Preventive measures include such as nutritional interventions with feed additives to improve gut health, strict nursery management, and rigorous biosecurity measures such as controlled movement of animals and handlers, dedicated equipment, and clean outer clothing [10,19,20]. Additional strategies, including age-based isolation and regular cleaning and disinfection, have been recognized as reducing pathogen spread and environmental contamination, particularly of water sources [21].

Taken together, these preventive strategies, which range from strict biosecurity on farms to broader food system policies and even personal precautions when traveling, emphasize the complex challenge posed by ETEC. However, more advanced efforts are limited effect without a deeper understanding of the bacterium’s pathogenesis. In this sense, addressing the pathophysiology of ETEC, particularly the mechanisms of its toxins and colonization factors, may guide the development of novel approaches for the treatment and control of these infections.

The present work focuses on the major virulence factors of ETEC, namely colonization factors and toxins. Both components are described for each host species, with particular emphasis on the toxins as well as, their classification, and structural characterization. More specifically, this study will address the main physiological alterations and adaptive mechanisms induced by two main enterotoxins, namely heat-stable toxin (ST) and heat-labile toxin (LT), in the three most affected systems: the gastrointestinal system, the enteric nervous system (ENS), and the immune system. By integrating bacterial colonization and gastrointestinal physiology, this work aims to emphasize the importance of mechanistic insights not only for advancing scientific understanding but also as a fundamental asset in safeguarding the global food system. In short, this manuscript integrates the physiological and biochemical changes induced by both types of ETEC enterotoxins in two affected species, offering new insights for preventive food safety strategies within the One Health framework.

## 2. Search Strategy and Selection Criteria

The research within this systematic review was intended to encompass a high volume and quality of work in concordance with the recent guidelines for reporting systematic reviews (PRISMA 20) [22]. Based on this, the several research topics were considered, mainly using PubMed and Google Scholar databases. It is also important to mention that the World Health Organization (WHO) website was also consulted. In addition, no time frame was established for searching these questions to ensure that no information would be lost. But it is important to mention that studies demonstrating novelty in this area of research were prioritized. Furthermore, this search strategy will be detailed according to the following three principal stages of searching.

In the first sections of the manuscript, namely in the Introduction and the part where the virulence factors were classified, several types of studies and renowned authors in the field were consulted to contribute robust insights on the topics of “ETEC infection”, “ETEC contamination sources” and the “structural and biochemical characteristics of the virulence factors”. In this section, the Universal Protein Resource (UniProt) Database was relevant to complement the information about the structural characterization of enterotoxins.

With regard to the main body of this review, the selected articles were subject to the main inclusion criterion, i.e., any type of study related to the interactions of ETEC enterotoxins in one of the three body systems (gastrointestinal, ENS, immune), whether in pigs or humans. In addition, the survey process was mainly carried out using some of the following keywords “ETEC enterotoxins”, “Heat-Labile toxin”, “Heat-Stable Toxin”, “ST”, “LT”, “human”, “pigs”, “main physiological interactions”, “gastrointestinal system”, “enteric nervous system”, and “immune system”. However, these searching process was performed mostly step by step, to ensure that the effect of each individual toxin (LT, ST) in each particular body system was explored.

In the final section, we also integrated relevant research regarding preventive measures in food processing and animal husbandry to ensure food safety practices, taking into account the farm-to-fork strategy. In addition, the limitations and future research priorities were highlighted to better extrapolate results from the physiological alterations induced by the ETEC-enterotoxins. These topics were researched separately using other keywords such as “ETEC contamination in foods”, “preventing bacterial contamination in foods”, “enteroid models”, “biomarkers of subclinical exposure” and “immune correlates of protection”.

Taken together, this information will contribute to empowering food science by providing in-depth knowledge about physiological changes induced by ETEC enterotoxins.

## 3. ETEC Virulence Factors: Fimbriae and Toxins

### 3.1. Classification of ETEC Colonization Factors

ETEC attachment to epithelial cells of the human small intestine occurs through different typologies of colonization factors (CFs), such as non-fimbrial, fimbrial, helical, or fibrillar. The traditional classification of ETEC colonization factors antigens (CFAs) groups them into three major families: CFA/I, CFA/II, and CFA/IV [23]. This nomenclature reflects the early characterization of ETEC CFs based on serological and phenotypic criteria. CFA/I is unique within its family, although subtypes have been identified. Strains expressing CFA/II encode coli surface antigen 3 (CS3), either alone or in combination with CS1 or CS2, while CFA/IV strains encode CS6, alone or combined with CS4 or CS5 [24,25]. However, subsequent molecular analyses have shown that these classical CFA groupings do not always correspond to the true genetic relatedness among CFs. Based on genomic, structural, and antigenic similarities, the 29 human ETEC CFs that have been identified so far are now divided into four main lineages: CFA/I, CS5, Class 1b group and a more diverse group of CFs (Table 1) [26].

Regarding the cognate receptors for these CFs, interactions of CFA/I with carbohydrate moieties of non-acid glycosphingolipids and glycoproteins, as well as CFA/IV with acid glycosphingolipid sulphatide, were described by Janson and colleagues in references [27,28], respectively. In addition, Roy and colleagues demonstrated that flagella that are transiently bound at the tip with the secreted adhesin EtpA can be used as epithelial-cell adherence factors [29]. Therefore, both CFs and flagella allow ETEC to anchor to enterocytes; nevertheless, more intimate attachment can be promoted by Tia and TibA63, known outer-membrane proteins [1,23].

Conversely to humans, with respect to swine-associated ETEC strains, the five common antigenically different types of CFs are F4 (K88), F5 (K99), F41, F6 (987P), and F18 [10,30]. The first four fimbria types are the ones that mediate adhesion in neonates, whereas F18 is only associated with postweaning diarrhea, in this case, together with the F4 type [6].

### 3.2. Classification and Structural Characterization of ETEC Enterotoxins

For both humans and pigs, ETEC releases two major classes of enterotoxins (Table 2), heat-labile enterotoxins (LTs) and heat-stable enterotoxins (STs), that interact with the enterocytes, thereby stimulating the secretion of water and electrolytes into the intestinal lumen [8]. The expression of these toxins in ETEC strains may occur individually (ST or LT) or together (ST + LT) and vary geographically and seasonally [31]. Also, most types of enterotoxins are plasmid encoded, and their nomenclature is based on toxin size, sequence, and biological activity [32].

#### 3.2.1. Heat-Labile Toxins (LT)

In respect to the LT class, it is closely related in structure and function to the enterotoxin expressed by *Vibrio cholerae*, known as cholera enterotoxin (CT) [33]. Both are part of an important group of toxins, the AB5 toxin family. This means that LT is composed of two subunits, A and B. The first consists of a single enzymatic polypeptide, and the second is a pentamer. Subunit A is non-covalently associated with the B subunit. The B subunit is the part that enables the binding with its respective receptor and the A subunit is the catalytically active component. The A subunit is composed of two fragments, A1 (ADP-ribosyl transferase) and A2 (peptide), which are bonded with a disulfide bond passing through a pore of the B subunit. The synthesis of A and B subunits occurs in the cytoplasm, while holotoxin structure processing occurs in the periplasm [31,34]. In addition, a study performed in 1980 reported that LT has an isoelectric point of pH 8.0 and can be dissociated into its A and B regions by a chaotropic reagent, such as guanidine [35]. LT can be divided into LT-I and LT-II, where the first one is found predominantly in human isolates and has ~80% amino acid identity with CT, whereas the second is found in some ETEC isolates from animals [2]. With regard to amino acid sequences, the A subunits of LT-I and LT-II are highly homologous, whereas the B subunits are markedly divergent between these subtypes [36,37,38]. The LT-I subtype is plasmid-encoded and can also be subdivided into LT-Ih and LT-Ip, which are produced by human- and porcine-associated strains, respectively [32]. The LT-II subtype includes the LT-IIa, LT-IIb and LT-IIc antigenic variants, which are encoded by chromosomal and prophage elements [39,40]. Regarding their host specificity, LT-IIa and LT-IIc are mainly predominant in water-buffalo/humans and humans/calves, respectively. Variant LT-IIb’s predominance in the host is still unknown [41].

#### 3.2.2. Heat-Stable Toxins (ST)

In the 1970s, STs attracted attention when it was observed that the heat inactivation process, intended to remove enterotoxigenic activity from bacterial cultures in diarrhea studies involving human and animal fecal samples, was ineffective [42,43]. Their small size, comprising fewer than 50 amino acids, along with their three-dimensional (3D) structure, confers resistance to boiling. Moreover, based on their sequences and biochemical characteristics, the following three types are recognized: STa (or ST-I), STb (or ST-II), and enteroaggregative *Escherichia coli* heat-stable enterotoxin 1 (EAST1) [32].

##### 3.2.2.1. STa Toxin

STa enterotoxinsare subdivided into STaP (porcine), which has been observed in isolates from several animal species (including humans) [44], and STaH (humans), which is solely produced by human isolates [32]. The active mature STa enterotoxin, composed of 18 or 19 amino acids for STaP and STaH, respectively, is generated by cleavage of its synthesized larger precursor, which contains 72 amino acids [45,46]. Both STaP and STaH contain six cysteine residues involved in disulfide bond formation, present in the same position. Additionally, the native form of these toxins, with their critical disulfide-bonded tertiary structure, is poorly immunogenic while maintaining their full biological activity [47]. This molecule is soluble in water and organic solvents, such as methanol, and is resistant to several proteases and acidic pH (in contrast to basic pH). Disruption of the disulfide bonds leads to toxin inactivation [32].

##### 3.2.2.2. STb Toxin

The STb enterotoxin is primarily isolated from pigs, but can also be sporadically found in other animal species, including cattle, chickens, dogs, cats, ferrets, and humans [48]. The active mature STb enterotoxin consists of 48 amino acids, with four cysteine residues involved in disulfide bridge formation. This active STb peptide is generated through the cleavage of its larger precursor, which is composed of 71 amino acids, including a 23-amino-acid signal sequence [49]. This toxin exhibits no homology with the STa or EAST1 enterotoxins. The biological activity is fully expressed in the peptide spanning from Cys10 to Cys48. STb is poorly immunogenic and a highly basic protein with an isoelectric point of 9.6 [50]. Additionally, it is soluble in water and certain organic solvents, but insoluble in methanol. The biological activity of STb is lost after treatment with β-mercaptoethanol or trypsin [42,51,52], and it also shows susceptibility to protease degradation [32,53].

##### 3.2.2.3. EAST1 Toxin

Another toxin of interest is EAST1, which exhibits certain physical and biological similarities to STa. However, unlike STa, EAST1 alone does not appear to cause disease [54], unless it is combined with the LT [55]. The gene encoding EAST1 has been detected in some diarrheagenic *E. coli* pathovars, including ETEC [56,57,58]. Nevertheless, this toxin was initially identified in an enteroaggregative *E. coli* (EAEC) strain isolated from the stool of a Chilean child with diarrhea [32,59,60]. Given that its role in pathogenesis is not yet fully understood, EAST1 will not be discussed further in the following sections.

## 4. Main Physiological Alterations and Adaptive Mechanisms

### 4.1. Gastrointestinal System (GI)

**Figure 1:**
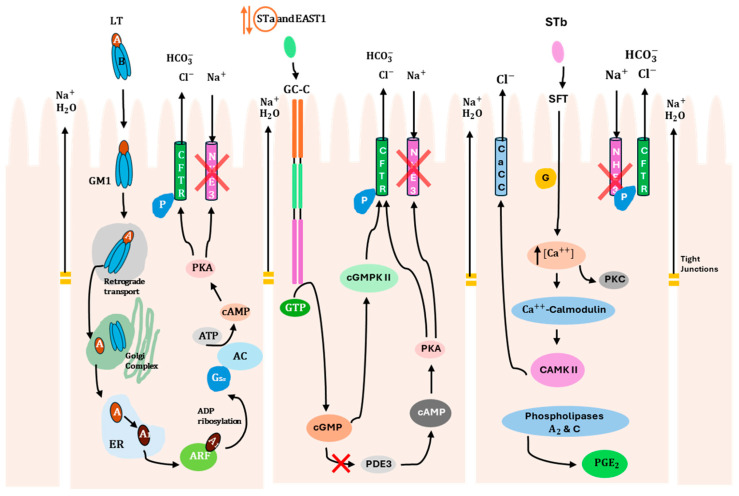
Mechanisms of internalization and action of ETEC enterotoxins (LT, STa/EAST1, STb) on intestinal epithelial cells. Description of specific molecular virulence pathways of each toxin that promote water and electrolyte loss through activation of ion channels and loosening of tight junctions. STa enterotoxin is surrounded by orange due to its reversible effect. The red symbol (X) indicates that Na^+^ reabsorption is inhibited in the epithelial cells. CFTR, cystic fibrosis transmembrane regulator; AC, adenylate cyclase; ARF, ADP-ribosylation factor; PKA, protein kinase A; PKC, protein kinase C; GM1, ganglioside GM1; GC-C, guanylate cyclase C; SFT, sulfatide; ER, endoplasmic reticulum; Gsα, α component of a heterotrimeric G protein; NHE3, Na+/H+-exchanger 3; PDE3, phosphodiesterase 3; cGMPKII, cGMP-dependent protein kinase II; cAMPKII, calmodulin-dependent protein kinase II; CaCC, calcium-activated chloride channel; P, phosphorylation.

ETEC produces two main types of enterotoxins (LT and ST), which are responsible for watery diarrhea in humans and pigs, as mentioned above. The basic molecular mechanisms underlying this illness are well-established [61]. In general, LT and ST activate the production of cyclic adenosine monophosphate (cAMP) and cyclic guanosine monophosphate (cGMP) second messengers, respectively, which then activate cellular kinases that modulate the activity of sodium and chloride channels in the apical membrane of intestinal epithelial cells, thereby promoting the net efflux of salt and water into the intestinal lumen [62]. Therefore, in this section, the mechanisms of internalization and action, as well as the epithelial receptors for each toxin, will be addressed individually to better understand the specific virulence pathways of these ETEC toxins (LT, STa, and STb) in the gastrointestinal tract (Figure 1). It is important to note that both humans and pigs share these virulence pathways, despite differences in the predominant classes of toxins.

With respect to LT, it is secreted from the bacterial cell [63] and interacts with lipopolysaccharide on the epithelial surface, facilitating adhesion [64]. The B subunit interacts with monosialoganglioside GM1 host receptor (Table 3), allowing toxin internalization at lipid rafts [65], where it is trafficked to the cytosol through the endoplasmic reticulum [1]. The A subunit modifies a specific host protein (stimulatory guanine-nucleotide α-subunit, G_sα_) by adding an ADP-ribose group. This modification activates the G protein, which activates the enzyme adenylyl cyclase, leading to an increase in intracellular cAMP. This activates cAMP-dependent protein kinase A (PKA), which triggers the activation of the cystic fibrosis transmembrane regulator (CFTR) chloride ion channel [4]. As a result, chloride ions efflux from the cell, leading to water movement into the intestinal lumen [31]. Additionally, the inhibition of antimicrobial peptide expression, which is part of the first line of immune defense [66], occurs through the activation of PKA and other host cell signaling pathways by LT [67].

In turn, STa toxin activates an intracellular signaling cascade through binding to a guanylate cyclase C (GC-C) receptor that belongs to the atrial natriuretic peptide receptor family [45]. This GC-C receptor is located on the villi of the jejunum and ileum, at the brush border of enterocytes [68]. After binding, GTP hydrolysis occurs, leading to cellular accumulation of cGMP due to the activation of the intracellular catalytic domain of GC-C [69]. Subsequently, cGMP-dependent protein kinase II (cGMPKII) is activated due to the high levels of cGMP, resulting in the phosphorylation of CFTR [70]. Activation of CFTR promotes the secretion of Cl^−^ and HCO_3_^−^, creating an osmotic gradient that draws water into the intestinal lumen, resulting in fluid secretion [71]. In addition, the elevated concentration of cGMP also inhibits phosphodiesterase 3 (PDE3), which causes an increase in cAMP levels, resulting in PKA activation. PKA has the dual function of phosphorylating both CFTR and Na^+^/H^+^-exchanger 3 (NHE3). As a result, phosphorylation of NHE3 inhibits Na^+^ reabsorption by the epithelial cells [45]. However, it is relevant to highlight that STa toxin has a reversible effect, meaning that after removal of the toxin, epithelial cells are able to recover their function [32]. It is also important to note that STa can reduce the integrity of tight junctions in epithelial cells. A study performed by [72] demonstrated that STa treatment of T84 polarized cell monolayers leads to a reduction in transepithelial resistance (TER) [32]. In addition, it is worth mentioning that this mechanism of STa internalization in enterocytes is shared by the EAST1 toxin.

Finally, STb binds through its galactose sulfate moiety to an acidic glycosphingolipid, which is highly abundant in the plasma membrane of epithelial cells. This binding stimulates a GTP-binding regulatory protein, leading to an increase in intracellular Ca^2+^ levels and activating calcium/calmodulin-dependent protein kinase II. As a consequence of the Ca^2+^ increase, protein kinase C (PKC) is activated, resulting in the phosphorylation of CFTR [73,74] and inhibition of Na^+^ uptake through an unidentified Na^+^ channel. Phosphorylation of CFTR can also be mediated by calcium/calmodulin-dependent protein kinase II, which activates a calcium-activated chloride channel (CaCC). Additionally, elevated intracellular Ca^2+^ levels influence the activity of phospholipases A2 and C, resulting in the release of arachidonic acid from membrane phospholipids and the production of prostaglandin E2 (PGE2) and serotonin (5-HT). These compounds mediate the transport of H_2_O and electrolytes out of the intestinal cells through an unknown mechanism [32].

#### Non-Diarrheal Sequelae Associated with ETEC Infections

With regard to the GI system, particular attention should also be directed to the non-diarrheal sequelae associated with ETEC infections. These include environmental enteric dysfunction (EED) and tropical sprue [75]. EED is a condition characterized by impaired nutrient absorption and growth [76,77], as well as malnutrition [78], all of which contribute to morbidity and, in some cases, mortality resulting from diarrhea or other infections [79]. Similar to EED, tropical sprue is linked to alterations in the small intestine’s villous structure, including changes in the ultrastructure of the epithelial brush border formed by the microvilli, as well as nutrient malabsorption and wasting [62].

**Figure 2:**
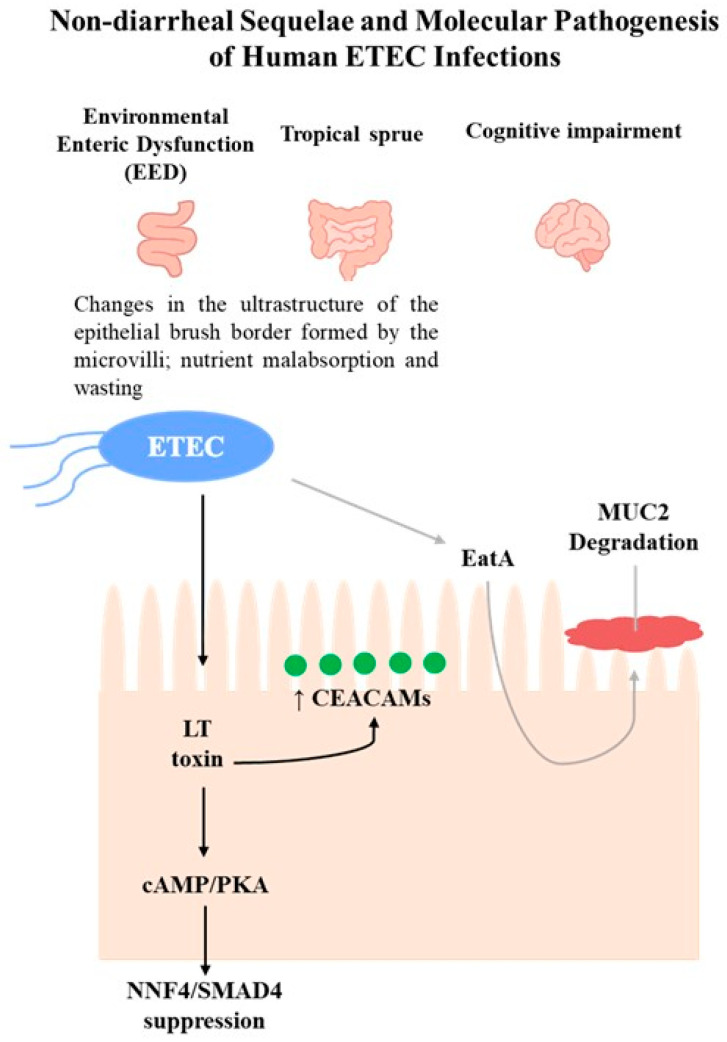
Schematic overview of non-diarrheal sequelae (EED, tropical sprue, cognitive impairment) and ETEC pathogenesis through LT (black arrows) and EatA (gray arrows) pathways. LT induces CEACAM overexpression and suppresses HNF4/SMAD4, while EatA promotes MUC2 degradation.

It is equally important to mention that ETEC infections have been associated with cognitive impairment sequelae [80,81]. However, the molecular mechanisms that cause these sequelae have only recently begun to be investigated, in contrast to those involved in the fluid and ion fluxes into the intestinal lumen that lead to diarrhea, which are well-established. Briefly, several studies provided evidence suggesting that ETEC may be responsible for previously unappreciated changes in the small intestinal epithelium (Figure 2) [62,82,83]. It was demonstrated that the LT induces transcriptional activation of genes encoding carcinoembryonic antigen-related cell adhesion molecules (CEACAM) expressed in intestinal epithelial cell cultures (Caco-2 cells, *in vitro*; human small intestinal enteroids, *ex vivo* human model). These molecules function as receptors for the type 1 fimbriae, chromosomally encoded by ETEC. The overexpression of CEACAMs may benefit the pathogen while being detrimental to the host, as it accelerates the processes of bacterial adhesion and toxin delivery. Therefore, to promote pathogen–host interactions, ETEC can effectively modulate the architecture of the small intestinal epithelium [82]. For example, the enhanced expression of CEACAMs has been shown to inhibit the anoikis process [84] and disrupt epithelial architecture, suggesting a link to enteropathy in ETEC infection. Anoikis is a natural process in which enterocytic cells that lose their connection to the underlying extracellular matrix are removed from the tips of the microvilli by apoptosis, which occurs during the typical maturation of these intestinal cells [85,86].

Recently, it was demonstrated that a secreted autotransporter protein, known as EatA, produced by certain ETEC strains, seems to be related to the development of symptomatic infection, according to molecular epidemiology studies [77,87]. Studies performed by Sheikh and coworkers showed that EatA plays a prominent role in the molecular pathogenesis of ETEC, as it degrades the MUC2 mucin barrier to promote bacterial access to target enterocytes and support toxin delivery, using human small intestine explants and small intestinal enteroids [83]. The function of MUC2 is to serve as the primary mucosal defense against GI pathogens and resident microbiota. It is secreted by goblet cells, assembling into large, layered polymeric net-like structures [88]. Interestingly, in contrast to EatA, LT has been shown to stimulate MUC2 production by goblet cells in the human small intestine, thereby enhancing the barrier between pathogens and intestinal cells [83].

The most recent study published by Sheikh and colleagues highlighted that the LT drives enteropathic changes in the small intestinal epithelium in both human small intestinal enteroids (*ex vivo* human model) and neonatal mouse models, through its typical mechanism of internalization into intestinal cells. As described in the previous section, LT increases cellular cAMP levels, activating PKA, which phosphorylates ion channels and generates electrolyte imbalances, leading to diarrhea [62]. Nonetheless, cAMP, through a number of cAMP-responsive transcriptional activators and repressors [89], can regulate various cellular processes [90] and modulate the transcription of multiple genes. The resultant activation of PKA leads to the dissociation of its two regulatory subunits from the catalytic subunits, which are then available to phosphorylate several protein substrates [91]. Therefore, PKA phosphorylates transcription factors such as cAMP response element-binding protein (CREB) and the cAMP-response element modulator (CREM). This phosphorylation enables the binding of these transcription factors to cAMP-response elements (CRE) in the promoter regions of target genes, thereby regulating transcription [89,91,92]. In this study, it was shown that LT significantly alters intestinal epithelial gene expression related to the biogenesis of the brush border, a critical region of the intestine responsible for the majority of nutrient absorption. Furthermore, LT suppresses the transcription factors HNF4 and SMAD4, which are essential for enterocyte differentiation and severely disrupts microvillus architecture and essential nutrient transport.

### 4.2. Enteric Nervous System (ENS)

The GI tract is unique among other hollow organs for possessing a complete nervous system that can function independently of neural inputs from the central nervous system (CNS), which includes the brain and spinal cord [93]. The first observation of this capacity was carried out by Von Haller, in 1755, who described the peristaltic reflex for the first time, which gave rise to the notion that the gut was capable of generating reflex responses independently of the CNS [94].

The ENS is composed of two distinct ganglionated neuronal plexuses: the myenteric and submucosal plexuses, which consist of thousands of discrete small ganglia that maintain neural continuity with each other [95]. The smooth muscle cells comprising the GI tract are densely innervated by both excitatory and inhibitory motor neurons [96,97,98]. Within each plexus, there exists a heterogeneous population of specific neurons with distinct neurochemical coding, projections, and functional roles [95,99,100]. In order to facilitate the rapid conduction of neuronal signals along the bowel, the ganglia within both plexuses are connected to neighboring ganglia via internodal strands that carry axons over substantial distances, up to 13 cm [101,102]. Regarding the function of each plexus, the myenteric plexus coordinates muscle movements underlying the propulsion of content, whereas the submucosal plexus is primarily involved in secretion and absorption [95]. The neurons that compose the ENS can be classified into intrinsic afferent neurons, interneurons, and motor secretory neurons [103].

Essentially, the ENS, through neurotransmitters such as substance P and acetylcholine, as well as the release of 5-hydroxytryptamine (5-HT) from enterochromaffin cells (EC), can partially mediate the intestinal secretion in response to luminal distension and feeding [104,105].

Infections caused by various bacterial and viral pathogens, including *V. cholerae*, ETEC, *Clostridium difficile*, and rotavirus, have been associated with the involvement of neural pathways [106,107]. It is important to note that many compounds secreted by bacteria, either alone or in combination, can activate afferent neurons in the villi [108]. Specifically, some secretory enterotoxins act as reflex arc activators by releasing endogenous mediators, such as 5-HT (or serotonin) and prostaglandins (PG) [103]. Nevertheless, in contrast to CT toxin, LT does not release 5-HT from enterochromaffin cells in the small intestine, as its secretory state is not inhibited by 5-HT receptor antagonists [109]. Additionally, LT-induced secretion is not inhibited by substance P antagonists. However, igmesine, a sigma receptor ligand, does inhibit LT secretion. Furthermore, the action of LT is inhibited by hexamethonium and lignocaine, further supporting the involvement of the ENS in LT secretion [110].

The ENS also appears to be involved in the secretory activity of ST, as its fluid secretion is inhibited by tetrodotoxin, lignocaine, and hexamethonium, as evaluated in *in vivo* animal models [111]. Similarly, to LT, the release of 5-HT from enterochromaffin cells does not appear to be implicated. Conversely, nitric oxide (NO) has been linked to ST-induced secretion, although neither tetrodotoxin nor nitric oxide synthase (NOS) inhibitor, such as L-NAME, interferes with ST-induced changes in short-circuit current in muscle-stripped preparations of pig jejunum and colon [110]. However, in addition to the studies conducted during the transition from the 20th to the 21st century, a more recent study, carried out in 2023 [112], aimed to evaluate the neural mechanisms associated with gastrointestinal secretory and motor functions in the pig colon, as well as to understand the functional disruptions induced by ETEC. Therefore, it was demonstrated that neurosecretion in swine is mediated by acetylcholine (ACh) and vasoactive intestinal peptide (VIP). In addition, the 5-HT3 and nicotine receptors are likely involved in the neural pathways associated with motility and secretion. In animals infected with ETEC, transient alterations in the barrier function and activation of neural mechanisms associated with increased secretion were observed. Furthermore, since mastocytosis was observed at the mucosal and submucosal levels, it is presumed that immunological mechanisms may also be involved in their activation. Unlike the neurosecretory mechanisms, neuromuscular mechanisms related to contraction and relaxation were not modified. Therefore, it was hypothesized that colonic motility is not affected by ETEC infection and that diarrhea is primarily a secretory defense mechanism involving the ENS [112].

From a translational perspective, GC-C and CFTR represent potential targets to counteract the effects of ETEC enterotoxins. For example, linaclotide (an orally administered drug for the treatment of constipation-predominant irritable bowel syndrome and chronic idiopathic constipation), a known GC-C agonist, has been shown to exhibit potent anti-nociceptive effects in several mechanistically distinct rodent models of visceral hypersensitivity [113]. This effect occurs through activation of the GC-C/cGMP pathway, which is also the typical mechanism underlying STa-ETEC infection. In addition, modulators targeting CFTR have also been explored as potential therapeutic tools. Since secretory diarrheas are caused by the hyperactivation of this channel, these modulators aim to downregulate its function. Two main types of therapeutic strategies have been developed: small-molecule CFTR inhibitors and macromolecular complexes. The small-molecule CFTR inhibitors include several naturally derived compounds, such as crofelemer, tannins, lysophosphatidic acid (LPA), and cocoa-related flavonoids, which act directly on the CFTR channel to reduce chloride secretion. In addition, therapeutic strategies targeting macromolecular complexes, particularly the CFTR/NHERF2/LPA2 complex in the plasma membrane of intestinal epithelial cells, have been identified. Modulation of this complex through CFTR-NHERF2 inhibitors or LPA2 agonists, including LPA itself, can downregulate CFTR channel function, providing a complementary approach to small-molecule inhibition [114].

### 4.3. Immune System

In addition to the interference of ETEC virulence factors in the GI system and ENS, intestinal cells produce inflammatory mediators that attract and activate innate immune cells, helping to coordinate immune responses to combat ETEC infections [115,116,117]. However, beyond its indirect effect on immune cells, the LT is a potent immunogen that activates dendritic cells, generating mucosal immune responses [118]. Remarkably, LT also reduces macrophage phagocytosis and mitigates inflammation by promoting the polarization of alternatively activated macrophages [119]. Regarding ST, in addition to being less studied, recent data indicate that the STa type does not directly influence macrophage function [41,119,120]. A study conducted by Loss and colleagues using *in vivo* piglet models demonstrated that ETEC infection triggers a broad antibacterial response in small intestinal tissues, characterized by the upregulation of pancreatitis-associated protein 1 (PAP), an antimicrobial peptide, matrix metalloproteinase 1 (MMP1), and the chemokine IL-8 [121]. Additionally, an STb-specific response was identified, involving matrix metallopeptidase 3 (MMP3) and immune-related genes encoding interleukins (IL), such as IL-17A, IL-1α, and IL-1β [121].

On the other hand, STa was found to induce intestinal secretion in the small intestine using a small intestinal segment perfusion (SISP) model [122], highlighting a functional impact of this toxin on intestinal physiology. However, despite this effect on intestinal function, the knowledge regarding the effect of these enterotoxins on the function of innate (neutrophils, macrophages) and adaptive immune cells (T and B cells) residing within the villus epithelium remains limited [41]. Nevertheless, more recently, Ma and colleagues evaluated the impact of LT and STa toxins on the effector functions of neutrophils in pig models [120]. Thus, according to their findings, LT reduced the phagocytic capacity of neutrophils, increased neutrophil migration and neutrophil extracellular trap (NET) formation and enhanced the secretion of IL-1β, CXCL-8, and TNF-α, whereas reactive oxygen species (ROS) production was not affected by this toxin. Also, it was demonstrated that the LT-induced migration and cytokine secretion by neutrophils were due to the activation of cAMP/PKA and ERK1/2 signaling pathways. In contrast, STa toxin did not affect neutrophil effector functions.

Finally, it is worth mentioning the double-mutant heat-labile toxin (dmLT), an LT derivative with two-point mutations, which has been used as a potent mucosal adjuvant with low enterotoxicity. Leach and colleagues in 2012 demonstrated that dmLT enhances IL-17A and IFN-γ responses *in vitro* in peripheral blood mononuclear cell (PBMC) cultures isolated from human volunteers immunized with ETEC vaccines [123]. More recently, this adjuvant (dmLT) has been validated in human clinical trials. In 2023, an oral inactivated ETEC vaccine (ETVAX^®^) formulated with dmLT was assessed for safety, tolerability and immunogenicity in healthy adults and children in Zambia (PACTR Ref. 201905764389804). This study reported strong plasma IgA and IgG responses against LTB in adults (100%) and children (80–90%) [124]. In addition, the EU-funded ETVAX project (EU Horizon 2020; Grant Agreement No. 778253) will conduct a phase II vaccine study to facilitate market approval of this vaccine for traveler’s diarrhea. Furthermore, in 2025, another trial (NCT03548064) evaluated the safety and immunogenicity of recombinant dmLT administered by three different routes (oral, sublingual, and intradermal) in healthy Bangladeshi adults. The study reported that dmLT was well tolerated and that immune responses depended on both the dose and route of administration [125].

## 5. Conclusions

This work emphasizes that a deeper understanding of the physiological and biochemical changes caused by ETEC enterotoxins is crucial to ensure food safety. By analyzing the complex responses of the gastrointestinal, enteric nervous and immune systems to ETEC infections, we have uncovered adaptive mechanisms that are shared by both pigs and humans, such as the regulation of intestinal motility, fluid balance and immune activation, which are essential for survival and recovery. The long-term consequences of ETEC exposure, including non-diarrheal outcomes such as EED and tropical sprue, emphasize the broader impact of these toxins on intestinal health and nutritional status, particularly in vulnerable populations.

Since ETEC infections in humans are mainly caused by animal handling or by consumption of farming associated products that are contaminated, like meat or fresh produce washed with unsanitary water, or improperly handled meat products, it is imperative that the work to control this type of infection begins upstream, in animal husbandry, and for this reason, the keeper/handler should, primarily, set up an animal health and welfare plan with the veterinarian responsible for the holding and, when necessary, with other technical consultants. This is not only a matter of animal health, per se, but also a guarantee of public health and prevention of contamination of all types of foods or water that serve both humans’ and animals’ needs. Also, preventive measures, including industry-driven reforms such as improved food handling protocols and consumer education programs, are extremely important to mitigate future ETEC-associated outbreaks. This control process needs to be aligned with the farm-to-fork strategy under the European Green Deal, enabling food systems to become fair, healthy, and environmentally friendly. Accordingly, across the entire food chain, it means that, from the primary sector to the market, sustainable practices in food production, processing, and consumption must be ensured, and effective measures should be implemented to prevent food loss and waste.

Finally, it is crucial to continue exploring the physiological interactions between ETEC and their enterotoxins and the affected systems, as this will provide valuable insights for the clinical management and prevention of ETEC-associated infections, as well as for the development of novel therapies in both human and veterinary medicine. Therefore, this knowledge will contribute to the preservation of public health, the reduction of antimicrobial use, and the lowering of the incidence of resistance genes.

Nevertheless, the scientific field still needs to evolve and develop novel approaches to overcome several challenges that limit reliable progress in understanding the complex molecular and dynamic mechanisms underlying ETEC infection in both pigs and humans, a transversal issue to several other gastrointestinal bacterial diseases. Therefore, first, to better mimic *in vivo* intestinal function, enteroid cultures should be standardized and further developed to include immune cells, stromal cells and microbiota interactions. In addition, identifying biomarkers of subclinical exposure is of prime importance to detect asymptomatic infections and to better understand naturally acquired immunity in endemic populations. Lastly, immune correlates of protection remain insufficiently defined, making it difficult to establish optimal treatment and vaccination strategies.

Advancing our knowledge of these host–pathogen interactions is crucial for understanding disease mechanisms and developing targeted prevention, treatment, and control strategies. Ultimately, this knowledge supports food safety by informing better clinical practices, reducing reliance on antimicrobials, limiting the spread of resistance genes and improving human and animal health outcomes.

## Figures and Tables

**Table 1:** Typical CFs of humans and pigs and their associated enterotoxins based on Mentzer et al., 2024 [26].

Colonization Factors (CFs)	Type of CF	Associated Enterotoxins	Host
**CFA/I-like group**			
CFA/I	Fimbrial	LT*, LT*+STaH, STaH	Human
CS1	Fimbrial	LT*+STaH	Human
CS2	Fimbrial	LT*+STaH	Human
CS4	Fimbrial	LT*+STp	Human
CS14	Fimbrial	LT*+STaH, STaH	Human
CS17	Fimbrial	LT*	Human
CS19	Fimbrial	LT*+STp	Human
PCFO71	Not determined		Human
**CS5-like group**			
CS5	Helical	LT*+STaH, STaH	Human
CS7	Helical	LT*+STaH	Human
**Class Ib group**			
CS12	Fimbrial	LT*+STp	Human
CS18	Fimbrial		Human
CS20	Fimbrial		Human
CS26	Not determined		Human
CS27A	Not determined	LT*, LT*+STp	Human
CS27B	Not determined	LT*, LT*+STp	Human
CS28A	Not determined	LT*, LT*+STp	Human
CS28B	Not determined	LT*, LT*+STp	Human
CS30	Fimbrial	LT*+STp	
F6	Fimbrial	LT*+STb	Neonatal piglets
**Diverse group**			
CS3	Fibrillar	LT*+STaH	Human
CS6	Non-fimbrial	LT*, LT*+STp, STp	Human
CS15	Non-fimbrial	Not determined	Human
CS22	Fibrillar	Not determined	Human
CS13 ^2^	Fibrillar	LT*, LT*+STp	Human
CS23 ^2^	Fibrillar/Non-fimbrial	Not determined	Human
F4	Fibrillar	LT*+STb, LT*+STaP+STb	Neonatal and weaned piglets
F41	Fibrillar	Not determined	Calves, lambs and goat kids, piglets
F5	Fibrillar	LT*+STb, STaP	Calves, lambs and goat kids
F18	Fimbrial	LT*+STb, STaP+STb	Weaned piglets
CS10 ^1^	Non-fimbrial	Not determined	Human
CS11 ^1^	Fibrillar	Not determined	Human
**Type IV pili**			
CS8 (CFA/III)	Fimbrial	LT*	Human
CS21	Fimbrial	LT*, LT*+STaH, STaH	Human

* LT can be either LT-Ih or LT-Ip. ^1^ Sequence and role of CS10 and CS11 are unknown. ^2^ Only ETEC lineages where most isolates share the same CF profiles are mentioned here.

**Table 2:** Structural characteristics (form; peptide sequence; aa; UniProt accession number) of toxins (STa, STb, LT-I and LT-II) produced by ETEC strains. East1 was not included in this table due to their absence of toxicity alone.

Toxins	Form	Peptide Sequence (N → C)	Length (aa)	UniProt Accession
STaH (human)	**Mature**	NSSNYCCELCCNPACTGCY	19	P07965
	**Precursor**	MKKSILFIFLSVLSFSPFAQDAKPVESSKEKITLESKKCNIAKKSNKSGPESMNSSNYCCELCCNPACTGCY	72	
STaP (porcine)	**Mature**	NTFYCCELCCNPACAGCY	18	P01559
	**Precursor**	MKKLMLAIFISVLSFPSFSQSTESLDSSKEKITLETKKCDVVKNNSEKKSENMNNTFYCCELCCNPACAGCY	72	
STb	**Mature**	STQSNKKDLCENYRQIAKESCKIGFLGVRDGTAGACFGAQIMVAAKGC	48	P22542
	**Precursor**	MKKNIAFLLASMFVFSIATNAYASTQSNKKDLCENYRQIAKESCKIGFLGVRDGTAGACFGAQIMVAAKGC	71	
LT-Ih A-subunit (human)	**Mature**	NGDKLYRADSRPPDEIKRSGGLMPRGHNEYFDRGTQMNINLYDHARGTQTGFVRYDDGYVSTSLSLRSAHLAGQSILSGYSTYYIYVIATAPNMFNVNDVLGVYSPHPYEQEVSALGGIPYSQIYGWYRVNFGVIDERLHRNREYRDRYYRNLNIAPAEDGYRLAGFPPDHQAWREEPWIHHAPQGCGNSSRTITGDTCNEETQNLSTIYLRKYQSKVKRQIFSDYQSEVDIYNRIRNEL	240	P43530
	**Precursor**	MKNITFIFFILLASPLYANGDKLYRADSRPPDEIKRSGGLMPRGHNEYFDRGTQMNINLYDHARGTQTGFVRYDDGYVSTSLSLRSAHLAGQSILSGYSTYYIYVIATAPNMFNVNDVLGVYSPHPYEQEVSALGGIPYSQIYGWYRVNFGVIDERLHRNREYRDRYYRNLNIAPAEDGYRLAGFPPDHQAWREEPWIHHAPQGCGNSSRTITGDTCNEETQNLSTIYLRKYQSKVKRQIFSDYQSEVDIYNRIRNEL	258	
LT-Ih B-subunit (human)	**Mature**	APQSITELCSEYHNTQIYTINDKILSYTESMAGKREMVIITFKSGATFQVEVPGSQHIDSQKKAIERMKDTLRITYLTETKIDKLCVWNNKTPNSIAAISMEN	103	P0CK94
	**Precursor**	MNKVKFYVLFTALLSSLCAHGAPQSITELCSEYHNTQIYTINDKILSYTESMAGKREMVIITFKSGATFQVEVPGSQHIDSQKKAIERMKDTLRITYLTETKIDKLCVWNNKTPNSIAAISMEN	124	
LT-Ip A-subunit (porcine)	**Mature**	NGDRLYRADSRPPDEIKRSGGLMPRGHNEYFDRGTQMNINLYDHARGTQTGFVRYDDGYVSTSLSLRSAHLAGQSILSGYSTYYIYVIATAPNMFNVNDVLGVYSPHPYEQEVSALGGIPYSQIYGWYRVNFGVIDERLHRNREYRDRYYRNLNIAPAEDGYRLAGFPPDHQAWREEPWIHHAPQGCGNSSRTITGDTCNEETQNLSTIYLREYQSKVKRQIFSDYQSEVDIYNRIRDEL	240	P06717
	**Precursor**	MKNITFIFFILLASPLYANGDRLYRADSRPPDEIKRSGGLMPRGHNEYFDRGTQMNINLYDHARGTQTGFVRYDDGYVSTSLSLRSAHLAGQSILSGYSTYYIYVIATAPNMFNVNDVLGVYSPHPYEQEVSALGGIPYSQIYGWYRVNFGVIDERLHRNREYRDRYYRNLNIAPAEDGYRLAGFPPDHQAWREEPWIHHAPQGCGNSSRTITGDTCNEETQNLSTIYLREYQSKVKRQIFSDYQSEVDIYNRIRDEL	258	
LT-Ip B-subunit (porcine)	**Mature**	APQTITELCSEYRNTQIYTINDKILSYTESMAGKREMVIITFKSGETFQVEVPGSQHIDSQKKAIERMKDTLRITYLTETKIDKLCVWNNKTPNSIAAISMKN	103	P32890
	**Precursor**	MNKVKCYVLFTALLSSLYAHGAPQTITELCSEYRNTQIYTINDKILSYTESMAGKREMVIITFKSGETFQVEVPGSQHIDSQKKAIERMKDTLRITYLTETKIDKLCVWNNKTPNSIAAISMKN	124	
LT-IIa A-subunit	**Mature**	NDFFRADSRTPDEIRRAGGLLPRGQQEAYERGTPININLYEHARGTVTGNTRYNDGYVSTTVTLRQAHLIGQNILGSYNEYYIYVVAPAPNLFDVNGVLGRYSPYPSENEFAALGGIPLSQIIGWYRVSFGAIEGGMQRNRDYRGDLFRGLTVAPNEDGYQLAGFPSNFPAWREMPWSTFAPEQCVPNNKEFKGGVCISATNVLSKYDLMNFKKLLKRRLALTFFMSEDDFIGVHGERDEL	241	P13810
	**Precursor**	MIKHVLLFFVFISFSVSANDFFRADSRTPDEIRRAGGLLPRGQQEAYERGTPININLYEHARGTVTGNTRYNDGYVSTTVTLRQAHLIGQNILGSYNEYYIYVVAPAPNLFDVNGVLGRYSPYPSENEFAALGGIPLSQIIGWYRVSFGAIEGGMQRNRDYRGDLFRGLTVAPNEDGYQLAGFPSNFPAWREMPWSTFAPEQCVPNNKEFKGGVCISATNVLSKYDLMNFKKLLKRRLALTFFMSEDDFIGVHGERDEL	259	
LT-IIa B-subunit	**Mature**	QVYAGVSEHFRNICNQTTADIVAGVQLKKYIADVNTNTRGIYVVSNTGGVWYIPGGRDYPDNFLSGEIRKTAMAAILSDTKVNLCAKTSSSPNHIWAMELDRES	104	P13812
	**Precursor**	MSSKKIIGAFVLMTGILSGQVYAGVSEHFRNICNQTTADIVAGVQLKKYIADVNTNTRGIYVVSNTGGVWYIPGGRDYPDNFLSGEIRKTAMAAILSDTKVNLCAKTSSSPNHIWAMELDRES	123	
LT-IIb A-subunit	**Mature**	NDYFRADSRTPDEVRRSGGLIPRGQDEAYERGTPININLYDHARGTATGNTRYNDGYVSTTTTLRQAHFLGQNMLGGYNEYYIYVVAAAPNLFDVNGVLGRYSPYPSENEYAALGGIPLSQIIGWYRVSFGAIEGGMHRNRDYRRDLFRGLSAAPNEDGYRIAGFPDGFPAWEEVPWREFAPNSCLPNNKASSDTTCASLTNKLSQHDLADFKKYIKRKFTLMTLLSINNDGFFSNNGGKDEL	243	P43528
	**Precursor**	MAKVISFFISLFLISFPLYANDYFRADSRTPDEVRRSGGLIPRGQDEAYERGTPININLYDHARGTATGNTRYNDGYVSTTTTLRQAHFLGQNMLGGYNEYYIYVVAAAPNLFDVNGVLGRYSPYPSENEYAALGGIPLSQIIGWYRVSFGAIEGGMHRNRDYRRDLFRGLSAAPNEDGYRIAGFPDGFPAWEEVPWREFAPNSCLPNNKASSDTTCASLTNKLSQHDLADFKKYIKRKFTLMTLLSINNDGFFSNNGGKDEL	263	
LT-IIb B-subunit	**Mature**	GASQFFKDNCNRTTASLVEGVELTKYISDINNNTDGMYVVSSTGGVWRISRAKDYPDNVMTAEMRKIAMAAVLSGMRVNMCASPASSPNVIWAIELEAE	99	P43529
	**Precursor**	MSFKKIIKAFVIMAALVSVQAHAGASQFFKDNCNRTTASLVEGVELTKYISDINNNTDGMYVVSSTGGVWRISRAKDYPDNVMTAEMRKIAMAAVLSGMRVNMCASPASSPNVIWAIELEAE	122	
LT-IIc A-subunit	**Mature**	NDFFRADTRTPSEIRQAGGLLPRGQQEAYERGTPININLYDHARGTVTGNTRYNDGYVSTTTTLRQAHLIGQNLLGSYNEYYIYVVAPAPNLFDVNGVLGRYSPYPSENEFAALGGIPLSQIIGWYRVSFGVIEGGMQRNRHYRRDLFQGLSVAPNHDGYHLAGFPDGFAAWRELPWSAFAPAACEHDYMVRILDACDSYTNRISKNDLFAFKRFMRIRSSLMILQSIEDDLQYNENKDEL	241	H6W8G4
	**Precursor**	MIKHLLLFFVFISFSVSANDFFRADTRTPSEIRQAGGLLPRGQQEAYERGTPININLYDHARGTVTGNTRYNDGYVSTTTTLRQAHLIGQNLLGSYNEYYIYVVAPAPNLFDVNGVLGRYSPYPSENEFAALGGIPLSQIIGWYRVSFGVIEGGMQRNRHYRRDLFQGLSVAPNHDGYHLAGFPDGFAAWRELPWSAFAPAACEHDYMVRILDACDSYTNRISKNDLFAFKRFMRIRSSLMILQSIEDDLQYNENKDEL	259	
LT-IIc B-subunit	**Mature**	GVSKTFKDNCASTTAKLVQSVQLVNISSDVNKDSKGIYISSSAGKTWFIPGGQYYPDNYLSNEMRKIAMAAVLSNVRVNLCASEAYTPNHVWAIELAP	98	H6W8G5
	**Precursor**	MNFKKLIALLFIVLNIASLPTYAGVSKTFKDNCASTTAKLVQSVQLVNISSDVNKDSKGIYISSSAGKTWFIPGGQYYPDNYLSNEMRKIAMAAVLSNVRVNLCASEAYTPNHVWAIELAP	121	

**Table 3:** Receptors of host cells (enterocytes) corresponding to each toxin (STa, EAST1, STb, LT-I, and LT-II) expressed by the ETEC strains based on Dubreuil and colleagues (2006) [32].

Toxin	Subtypes	Receptor(s)
STa	STaH	Guanylate cyclase C
	STaP	Guanylate cyclase C
EAST1		Guanylate cyclase C
STb		Sulfatide
LT-I	LT-Ih	^a^ GM1, GD1b, GM2, asialo GM1, galactoproteins, galactose-containing glycolipids
	LT-Ip	
LT-II	LT-IIa	^a^ GD1b, GD1a, GT1b, GQ1b, GD2
	LT-IIb	^a^ GD1a, GT1b, GM3
	LT-IIc	^a^ GM1, GM2, GM3, GD1a

^a^ In order of decreasing binding strength.

## Data Availability

No new data were created or analyzed in this study. Data sharing is not applicable to this article.

## References

[1] Croxen M.A., Finlay B.B. (2010). Molecular Mechanisms of *Escherichia coli* Pathogenicity. Nat. Rev. Microbiol..

[2] Kaper J.B., Nataro J.P., Mobley H.L.T. (2004). Pathogenic *Escherichia coli*. Nat. Rev. Microbiol..

[3] Sapountzis P., Segura A., Desvaux M., Forano E. (2020). An Overview of the Elusive Passenger in the Gastrointestinal Tract of Cattle: The Shiga Toxin Producing *Escherichia coli*. Microorganisms.

[4] Nataro J.P., Kaper J.B. (1998). Diarrheagenic *Escherichia coli*. Clin. Microbiol. Rev..

[5] World Health Organization (2021). WHO Preferred Product Characteristics for Vaccines Against Enterotoxigenic Escherichia coli.

[6] Barros M.M., Castro J., Araújo D., Campos A.M., Oliveira R., Silva S., Outor-Monteiro D., Almeida C. (2023). Swine Colibacillosis: Global Epidemiologic and Antimicrobial Scenario. Antibiotics.

[7] Luppi A. (2017). Swine Enteric Colibacillosis: Diagnosis, Therapy and Antimicrobial Resistance. Porc. Health Manag..

[8] Fairbrother J.M., Nadeau É., Zimmerman J.J., Karriker L.A., Ramirez A., Schwartz K.J., Stevenson G.W., Zhang J. (2019). Colibacillosis. Diseases of Swine.

[9] Fairbrother J.M., Nadeau É., Gyles C.L. (2005). *Escherichia coli* in Postweaning Diarrhea in Pigs: An Update on Bacterial Types, Pathogenesis, and Prevention Strategies. Anim. Health Res. Rev..

[10] Castro J., Barros M.M., Araújo D., Campos A.M., Oliveira R., Silva S., Almeida C. (2022). Swine Enteric Colibacillosis: Current Treatment Avenues and Future Directions. Front. Vet. Sci..

[11] Gyles C.L., Fairbrother J.M., Gyles C.L., Prescott J.F., Songer J.G., Thoen C.O. (2010). Escherichia coli. Pathogenesis of Bacterial Infections in Animals.

[12] Zhang Y., Tan P., Zhao Y., Ma X. (2022). Enterotoxigenic *Escherichia coli*: Intestinal Pathogenesis Mechanisms and Colonization Resistance by Gut Microbiota. Gut Microbes.

[13] Chatterjee A., Abraham J. (2018). Microbial Contamination, Prevention, and Early Detection in Food Industry. Handbook of Food Bioengineering.

[14] Urban-Chmiel R., Osek J., Wieczorek K. (2025). Methods of Controlling Microbial Contamination of Food. Pathogens.

[15] Ajekiigbe V.O., Ogieuhi I.J., Anthony C.S., Bakare I.S., Anyacho S., Ogunleke P.O., Fatokun D.I., Akinmeji O., Ruth O.T., Olaore A.K. (2025). Consumer Behavior and Its Role in *E. coli* Outbreaks: The Impact of Fast-Food Preparation Practices and Hygiene Awareness. Trop. Med. Health.

[16] Vázquez-Quiñones C.R., Rincón-Guevara M., Natividad-Bonifacio I., Vázquez-Salinas C., González-Márquez H. (2025). Incidence Rates of Resistant Enterotoxigenic *Escherichia coli* in Fresh Vegetables and Salads. Access Microbiol..

[17] Youmans B.P., Ajami N.J., Jiang Z.D., Campbell F., Duncan Wadsworth W., Petrosino J.F., Du-Pont H.L., Highlander S.K. (2015). Characterization of the Human Gut Microbiome during Travelers’ Diarrhea. Gut Microbes.

[18] Suffredini E., Pepe T., Ventrone I., Croci L. (2011). Norovirus Detection in Shellfish Using Two Real-Time RT-PCR Methods. New Microbiol..

[19] Amass S.F., Halbur P.G., Byrne B.A., Schneider J.L., Koons C.W., Cornick N., Ragland D. (2003). Mechanical Transmission of Enterotoxigenic *Escherichia coli* to Weaned Pigs by People, and Biosecurity Procedures That Prevented Such Transmission. J. Swine Health Prod..

[20] Kim K., Song M., Liu Y., Ji P. (2022). Enterotoxigenic *Escherichia coli* Infection of Weaned Pigs: Intestinal Challenges and Nutritional Intervention to Enhance Disease Resistance. Front. Immunol..

[21] Laird T.J., Abraham S., Jordan D., Pluske J.R., Hampson D.J., Trott D.J., O’Dea M. (2021). Porcine Enterotoxigenic *Escherichia coli*: Antimicrobial Resistance and Development of Microbial-Based Alternative Control Strategies. Vet. Microbiol..

[22] Page M.J., McKenzie J.E., Bossuyt P.M., Boutron I., Hoffmann T.C., Mulrow C.D., Shamseer L., Tetzlaff J.M., Akl E.A., Brennan S.E. (2021). The PRISMA 2020 Statement: An Updated Guideline for Reporting Systematic Reviews. BMJ.

[23] Turner S.M., Scott-Tucker A., Cooper L.M., Henderson I.R. (2006). Weapons of Mass Destruction: Virulence Factors of the Global Killer Enterotoxigenic *Escherichia coli*. FEMS Microbiol. Lett..

[24] Vidal R.M., Muhsen K., Tennant S.M., Svennerholm A.M., Sow S.O., Sur D., Zaidi A.K.M., Faruque A.S.G., Saha D., Adegbola R. (2019). Colonization Factors among Enterotoxigenic *Escherichia coli* Isolates from Children with Moderate-to-Severe Diarrhea and from Matched Controls in the Global Enteric Multicenter Study (GEMS). PLoS Negl. Trop. Dis..

[25] Levine M.M. (1987). *Escherichia coli* That Cause Diarrhea: Enterotoxigenic, Enteropathogenic, Enteroinvasive, Enterohemorrhagic, and Enteroadherent. J. Infect. Dis..

[26] von Mentzer A., Svennerholm A.-M. (2024). Colonization Factors of Human and Animal-Specific Enterotoxigenic *Escherichia coli* (ETEC). Trends Microbiol..

[27] Jansson L., Tobias J., Lebens M., Svennerholm A.-M., Teneberg S. (2006). The Major Subunit, CfaB, of Colonization Factor Antigen I from Enterotoxigenic *Escherichia coli* is a Glycosphingolipid Binding Protein. Infect. Immun..

[28] Jansson L., Tobias J., Jarefjäll C., Lebens M., Svennerholm A.-M., Teneberg S. (2009). Sulfatide Recognition by Colonization Factor Antigen CS6 from Enterotoxigenic *Escherichia coli*. PLoS ONE.

[29] Roy K., Hilliard G.M., Hamilton D.J., Luo J., Ostmann M.M., Fleckenstein J.M. (2009). Enterotoxigenic *Escherichia coli* EtpA Mediates Adhesion between Flagella and Host Cells. Nature.

[30] Hartadi E.B., Effendi M.H., Plumeriastuti H., Sofiana E.D., Wibisono F.M., Hidayatullah A.R. (2020). A Review of Enterotoxigenic *Escherichia coli* Infection in Piglets: Public Health Importance. Syst. Rev. Pharm..

[31] Read L.T., Hahn R.W., Thompson C.C., Bauer D.L., Norton E.B., Clements J.D. (2014). Simultaneous Exposure to *Escherichia coli* Heat-Labile and Heat-Stable Enterotoxins Increases Fluid Secretion and Alters Cyclic Nucleotide and Cytokine Production by Intestinal Epithelial Cells. Infect. Immun..

[32] Dubreuil J.D., Isaacson R.E., Schifferli D.M. (2016). Animal Enterotoxigenic *Escherichia coli*. EcoSalPlus.

[33] Spangler B.D. (1992). Structure and Function of Cholera Toxin and the Related *Escherichia coli* Heat-Labile Enterotoxin. Microbiol. Rev..

[34] Serrano A., Guyette J.L., Heim J.B., Taylor M., Cherubin P., Krengel U., Teter K., Tatulian S.A. (2022). Holotoxin Disassembly by Protein Disulfide Isomerase is Less Efficient for *Escherichia coli* Heat-Labile Enterotoxin than Cholera Toxin. Sci. Rep..

[35] Clements J.D., Yancey R.J., Finkelstein R.A. (1980). Properties of Homogeneous Heat-Labile Enterotoxin from *Escherichia coli*. Infect. Immun..

[36] Connell T.D. (2007). Cholera Toxin, LT-I, LT-IIa, and LT-IIb: The Critical Role of Ganglioside-Binding in Immunomodulation by Type I and Type II Heat-Labile Enterotoxins. Expert. Rev. Vaccines.

[37] Mekalanos J.J., Sublett R.D., Romig W.R. (1979). Genetic Mapping of Toxin Regulatory Mutations in *Vibrio cholerae*. J. Bacteriol..

[38] Duan Q., Xia P., Nandre R., Zhang W., Zhu G. (2019). Review of Newly Identified Functions Associated with the Heat-Labile Toxin of Enterotoxigenic *Escherichia coli*. Front. Cell. Infect. Microbiol..

[39] Nawar H.F., Berenson C.S., Hajishengallis G., Takematsu H., Mandell L., Clare R.L., Connell T.D. (2010). Binding to Gangliosides Containing N-Acetylneuraminic Acid is Sufficient to Mediate the Immunomodulatory Properties of the Nontoxic Mucosal Adjuvant LT-IIb(T13I). Clin. Vaccine Immunol..

[40] Casey T.A., Connell T.D., Holmes R.K., Whipp S.C. (2012). Evaluation of Heat-Labile Enterotoxins Type IIa and Type IIb in the Pathogenicity of Enterotoxigenic *Escherichia coli* for Neonatal Pigs. Vet. Microbiol..

[41] Wang H., Zhong Z., Luo Y., Cox E., Devriendt B. (2019). Heat-Stable Enterotoxins of Enterotoxigenic *Escherichia coli* and Their Impact on Host Immunity. Toxins.

[42] Burgess M.N., Bywater R.J., Cowley C.M., Mullan N.A., Newsome P.M. (1978). Biological Evaluation of a Methanol-Soluble, Heat-Stable *Escherichia coli* Enterotoxin in Infant Mice, Pigs, Rabbits, and Calves. Infect. Immun..

[43] Smith H.W., Gyles C.L. (1970). The Effect of Cell-Free Fluids Prepared from Cultures of Human and Animal Enteropathogenic Strains of *Escherichia coli* on Ligated Intestinal Segments of Rabbits and Pigs. J. Med. Microbiol..

[44] Nair G.B., Takeda Y. (1998). The Heat-Stable Enterotoxins. Microb. Pathog..

[45] Weiglmeier P.R., Rösch P., Berkner H. (2010). Cure and Curse: *E. coli* Heat-Stable Enterotoxin and Its Receptor Guanylyl Cyclase C. Toxins.

[46] Lima A.A.M., Fonteles M.C. (2014). From *Escherichia coli* Heat-Stable Enterotoxin to Mammalian Endogenous Guanylin Hormones. Braz. J. Med. Biol. Res..

[47] Okamoto K., Okamoto K., Yukitake J., Kawamoto Y., Miyama A. (1987). Substitutions of Cysteine Residues of *Escherichia coli* Heat-Stable Enterotoxin by Oligonucleotide-Directed Mutagenesis. Infect. Immun..

[48] Dubreuil J.D. (1997). *Escherichia coli* STb Enterotoxin. Microbiology.

[49] Lee C.H., Moseley S.L., Moon H.W., Whipp S.C., Gyles C.L., So M. (1983). Characterization of the Gene Encoding Heat-Stable Toxin II and Preliminary Molecular Epidemiological Studies of Enterotoxigenic *Escherichia coli* Heat-Stable Toxin II Producers. Infect. Immun..

[50] Handl C.E., Harel J., Flock J.I., Dubreuil J.D. (1993). High Yield of Active STb Enterotoxin from a Fusion Protein (MBP-STb) Expressed in *Escherichia coli*. Protein Expr. Purif..

[51] Dubreuil J.D., Fairbrother J.M., Lallier R., Lariviere S. (1991). Production and Purification of Heat-Stable Enterotoxin b from a Porcine *Escherichia coli* Strain. Infect. Immun..

[52] Fujii Y., Hayashi M., Hitotsubashi S., Fuke Y., Yamanaka H., Okamoto K. (1991). Purification and Characterization of *Escherichia coli* Heat-Stable Enterotoxin II. J. Bacteriol..

[53] Whipp S.C. (1987). Protease Degradation of *Escherichia coli* Heat-Stable, Mouse-Negative, Pig-Positive Enterotoxin. Infect. Immun..

[54] Ngeleka M., Pritchard J., Appleyard G., Middleton D.M., Fairbrother J.M. (2003). Isolation and Association of *Escherichia coli* AIDA-I/STb, Rather than EAST1 Pathotype, with Diarrhea in Piglets and Antibiotic Sensitivity of Isolates. J. Vet. Diagn. Investig..

[55] Berberov E.M., Zhou Y., Francis D.H., Scott M.A., Kachman S.D., Moxley R.A. (2004). Relative Importance of Heat-Labile Enterotoxin in the Causation of Severe Diarrheal Disease in the Gnotobiotic Piglet Model by a Strain of Enterotoxigenic *Escherichia coli* That Produces Multiple Enterotoxins. Infect. Immun..

[56] Ménard L.-P., Dubreuil J.D. (2002). Enteroaggregative *Escherichia coli* Heat-Stable Enterotoxin 1 (EAST1): A New Toxin with an Old Twist. Crit. Rev. Microbiol..

[57] Paiva de Sousa C., Dubreuil J.D. (2001). Distribution and Expression of the *astA* Gene (EAST1 Toxin) in *Escherichia coli* and Salmonella. Int. J. Med. Microbiol..

[58] Savarino S.J., McVeigh A., Watson J., Cravioto A., Molina J., Echeverria P., Bhan M.K., Levine M.M., Fasano A. (1996). Enteroaggregative *Escherichia coli* Heat-Stable Enterotoxin Is Not Restricted to Enteroaggregative *E. coli*. J. Infect. Dis..

[59] Savarino S.J., Fasano A., Robertson D.C., Levine M.M. (1991). Enteroaggregative *Escherichia coli* Elaborate a Heat-Stable Enterotoxin Demonstrable in an in Vitro Rabbit Intestinal Model. J. Clin. Investig..

[60] Levine M.M., Prado V., Robins-Browne R., Lior H., Kaper J.B., Moseley S.L., Gicquelais K., Nataro J.P., Vial P., Tall B. (1988). Use of DNA Probes and HEp-2 Cell Adherence Assay to Detect Diarrheagenic *Escherichia coli*. J. Infect. Dis..

[61] Fleckenstein J.M., Hardwidge P.R., Munson G.P., Rasko D.A., Sommerfelt H., Steinsland H. (2010). Molecular Mechanisms of Enterotoxigenic *Escherichia coli* Infection. Microbes Infect..

[62] Sheikh A., Tumala B., Vickers T.J., Martin J.C., Rosa B.A., Sabui S., Basu S., Simoes R.D., Mitreva M., Storer C. (2022). Enterotoxigenic *Escherichia coli* Heat-Labile Toxin Drives Enteropathic Changes in Small Intestinal Epithelia. Nat. Commun..

[63] Dorsey F.C., Fischer J.F., Fleckenstein J.M. (2006). Directed Delivery of Heat-Labile Enterotoxin by Enterotoxigenic *Escherichia coli*. Cell. Microbiol..

[64] Johnson A.M., Kaushik R.S., Francis D.H., Fleckenstein J.M., Hardwidge P.R. (2009). Heat-Labile Enterotoxin Promotes *Escherichia coli* Adherence to Intestinal Epithelial Cells. J. Bacteriol..

[65] Kesty N.C., Mason K.M., Reedy M., Miller S.E., Kuehn M.J. (2004). Enterotoxigenic *Escherichia coli* Vesicles Target Toxin Delivery into Mammalian Cells. EMBO J..

[66] Duarte-Mata D.I., Salinas-Carmona M.C. (2023). Antimicrobial Peptides’ Immune Modulation Role in Intracellular Bacterial Infection. Front. Immunol..

[67] Chakraborty K., Ghosh S., Koley H., Mukhopadhyay A.K., Ramamurthy T., Saha D.R., Mukhopadhyay D., Roychowdhury S., Hamabata T., Takeda Y. (2008). Bacterial Exotoxins Downregulate Cathelicidin (HCAP-18/LL-37) and Human Beta-Defensin 1 (HBD-1) Expression in the Intestinal Epithelial Cells. Cell Microbiol..

[68] Krause W.J., Cullingford G.L., Freeman R.H., Eber S.L., Richardson K.C., Fok K.F., Currie M.G., Forte L.R. (1994). Distribution of Heat-Stable Enterotoxin/Guanylin Receptors in the Intestinal Tract of Man and Other Mammals. J. Anat..

[69] Akabas M.H. (2000). Cystic Fibrosis Transmembrane Conductance Regulator. Structure and Function of an Epithelial Chloride Channel. J. Biol. Chem..

[70] Arshad N., Visweswariah S.S. (2012). The Multiple and Enigmatic Roles of Guanylyl Cyclase C in Intestinal Homeostasis. FEBS Lett..

[71] Hug M.J., Tamada T., Bridges R.J. (2003). CFTR and Bicarbonate Secretion to Epithelial Cells. News Physiol. Sci..

[72] Nakashima R., Kamata Y., Nishikawa Y. (2013). Effects of *Escherichia coli* Heat-Stable Enterotoxin and Guanylin on the Barrier Integrity of Intestinal Epithelial T84 Cells. Vet. Immunol. Immunopathol..

[73] Dreyfus L.A., Harville B., Howard D.E., Shaban R., Beatty D.M., Morris S.J. (1993). Calcium Influx Mediated by the *Escherichia coli* Heat-Stable Enterotoxin B (STB). Proc. Natl. Acad. Sci. USA.

[74] Fujii Y., Nomura T., Yamanaka H., Okamoto K. (1997). Involvement of Ca^2+^ -Calmodulin-Dependent Protein Kinase II in the Intestinal Secretory Action of *Escherichia coli* Heat-Stable Enterotoxin II. Microbiol. Immunol..

[75] Klipstein F.A., Holdeman L.V., Corcino J.J., Moore W.E.C. (1973). Enterotoxigenic Intestinal Bacteria in Tropical Sprue. Ann. Intern. Med..

[76] Nasrin D., Blackwelder W.C., Sommerfelt H., Wu Y., Farag T.H., Panchalingam S., Biswas K., Saha D., Jahangir Hossain M., Sow S.O. (2021). Pathogens Associated with Linear Growth Faltering in Children With Diarrhea and Impact of Antibiotic Treatment: The Global Enteric Multicenter Study. J. Infect. Dis..

[77] Qadri F., Saha A., Ahmed T., Al Tarique A., Begum Y.A., Svennerholm A.-M. (2007). Disease Burden Due to Enterotoxigenic *Escherichia coli* in the First 2 Years of Life in an Urban Community in Bangladesh. Infect. Immun..

[78] Anderson J.D., Bagamian K.H., Muhib F., Amaya M.P., Laytner L.A., Wierzba T., Rheingans R. (2019). Burden of Enterotoxigenic *Escherichia coli* and *Shigella* Non-Fatal Diarrhoeal Infections in 79 Low-Income and Lower Middle-Income Countries: A Modelling Analysis. Lancet Glob. Health.

[79] Caulfield L.E., de Onis M., Blössner M., Black R.E. (2004). Undernutrition as an Underlying Cause of Child Deaths Associated with Diarrhea, Pneumonia, Malaria, and Measles. Am. J. Clin. Nutr..

[80] MAL-ED Network Investigators (2018). Early Childhood Cognitive Development is Affected by Interactions among Illness, Diet, Enteropathogens and the Home Environment: Findings from the MAL-ED Birth Cohort Study. BMJ Glob. Health.

[81] Guerrant R.L., Deboer M.D., Moore S.R., Scharf R.J., Lima A.A.M. (2012). The Impoverished Gut—A Triple Burden of Diarrhoea, Stunting and Chronic Disease. Nat. Rev. Gastroenterol. Hepatol..

[82] Sheikh A., Tumala B., Vickers T.J., Alvarado D., Ciorba M.A., Bhuiyan R., Qadri F., Singer B.B., Fleckenstein J.M. (2020). CEACAMs Serve as Toxin-Stimulated Receptors for Enterotoxigenic *Escherichia coli*. Proc. Natl. Acad. Sci. USA.

[83] Sheikh A., Wangdi T., Vickers T.J., Aaron B., Palmer M., Miller M.J., Kim S., Herring C., Simoes R., Crainic J.A. (2022). Enterotoxigenic *Escherichia coli* Degrades the Host MUC2 Mucin Barrier to Facilitate Critical Pathogen-Enterocyte Interactions in Human Small Intestine. Infect. Immun..

[84] Ilantzis C., DeMarte L., Screaton R.A., Stanners C.P. (2002). Deregulated Expression of the Human Tumor Marker CEA and CEA Family Member CEACAM6 Disrupts Tissue Architecture and Blocks Colonocyte Differentiation. Neoplasia.

[85] Taddei M.L., Giannoni E., Fiaschi T., Chiarugi P. (2012). Anoikis: An Emerging Hallmark in Health and Diseases. J. Pathol..

[86] Barker N. (2014). Adult Intestinal Stem Cells: Critical Drivers of Epithelial Homeostasis and Regeneration. Nat. Rev. Mol. Cell Biol..

[87] Kuhlmann F.M., Laine R.O., Afrin S., Nakajima R., Akhtar M., Vickers T., Parker K., Nizam N.N., Grigura V., Goss C.W. (2021). Contribution of Noncanonical Antigens to Virulence and Adaptive Immunity in Human Infection with Enterotoxigenic *E. coli*. Infect. Immun..

[88] Johansson M.E.V., Phillipson M., Petersson J., Velcich A., Holm L., Hansson G.C. (2008). The Inner of the Two Muc2 Mucin-Dependent Mucus Layers in Colon is Devoid of Bacteria. Proc. Natl. Acad. Sci. USA.

[89] Sassone-Corsi P. (1995). Transcription Factors Responsive to CAMP. Annu. Rev. Cell Dev. Biol..

[90] Lodish H., Berk A., Matsudaira P., Kaiser C.A., Krieger M., Scott M.P., Zipursky L., Darnell J. (2000). Molecular Cell Biology.

[91] Sassone-Corsi P. (2012). The Cyclic AMP Pathway. Cold Spring Harb. Perspect. Biol..

[92] Zambon A.C., Zhang L., Minovitsky S., Kanter J.R., Prabhakar S., Salomonis N., Vranizan K., Dubchak I., Conklin B.R., Insel P.A. (2005). Gene Expression Patterns Define Key Transcriptional Events in Cell-Cycle Regulation by CAMP and Protein Kinase A. Proc. Natl. Acad. Sci. USA.

[93] Spencer N.J., Hu H. (2020). Enteric Nervous System: Sensory Transduction, Neural Circuits and Gastrointestinal Motility. Nat. Rev. Gastroenterol. Hepatol..

[94] Haller V. (1755). A Dissertation on the Sensible and Irritable Parts of Animals.

[95] Furness J.B. (2006). The Enteric Nervous System.

[96] Bennett M.R., Burnstock G., Holman M.E. (1966). Transmission from Perivascular Inhibitory Nerves to the Smooth Muscle of the Guinea-Pig Taenia Coli. J. Physiol..

[97] Bülbring E., Tomita T. (1967). Properties of the Inhibitory Potential of Smooth Muscle as Observed in the Response to Field Stimulation of the Guinea-Pig Taenia Coli. J. Physiol..

[98] Furness J.B. (2000). Types of Neurons in the Enteric Nervous System. J. Auton. Nerv. Syst..

[99] Costa M., Furness J.B., Gibbins I.L. (1986). Chemical Coding of Enteric Neurons. Prog. Brain Res..

[100] Costa M., Brookes S.J.H., Steele P.A., Gibbins I., Burcher E., Kandiah C.J. (1996). Neurochemical Classification of Myenteric Neurons in the Guinea-Pig Ileum. Neuroscience.

[101] Brookes S.J.H., Song Z.M., Ramsay G.A., Costa M. (1995). Long Aboral Projections of Dogiel Type II, AH Neurons within the Myenteric Plexus of the Guinea Pig Small Intestine. J. Neurosci..

[102] Furness J.B., Kunze W.A., Bertrand P.P., Clerc N., Bornstein J.C. (1998). Intrinsic Primary Afferent Neurons of the Intestine. Prog. Neurobiol..

[103] Dubreuil J.D. (2012). The Whole Shebang: The Gastrointestinal Tract, *Escherichia coli* Enterotoxins and Secretion. Curr. Issues Mol. Biol..

[104] Jodal M. (1990). Neuronal Influence on Intestinal Transport. J. Intern. Med..

[105] Cooke H.J. (2006). Neurotransmitters in Neuronal Reflexes Regulating Intestinal Secretion. Ann. N. Y. Acad. Sci..

[106] Pothoulakis C. (2000). The Role of Neuroenteric Hormones in Intestinal Infectious Diseases. Curr. Opin. Gastroenterol..

[107] Morris A.P., Estes M.K. (2001). Microbes and Microbial Toxins: Paradigms for Microbial-Mucosal Interactions. VIII. Pathological Consequences of Rotavirus Infection and Its Enterotoxin. Am. J. Physiol. Gastrointest. Liver Physiol..

[108] Kirkup A.J., Brunsden A.M., Grundy D. (2001). Receptors and Transmission in the Brain-Gut Axis: Potential for Novel Therapies. I. Receptors on Visceral Afferents. Am. J. Physiol. Gastrointest. Liver Physiol..

[109] Eherer A.J., Hinterleitner T.A., Petritsch W., Holzer-Petsche U., Beubler E., Krejs G.J. (1994). Effect of 5-Hydroxytryptamine Antagonists on Cholera Toxin-Induced Secretion in the Human Jejunum. Eur. J. Clin. Investig..

[110] Farthing M.J.G. (2000). Enterotoxins and the Enteric Nervous System—A Fatal Attraction. Int. J. Med. Microbiol..

[111] Eklund S., Jodal M., Lundgren O. (1985). The Enteric Nervous System Participates in the Secretory Response to the Heat Stable Enterotoxins of *Escherichia coli* in Rats and Cats. Neuroscience.

[112] Traserra S., Casabella-Ramón S., Vergara P., Jimenez M. (2023). *E. coli* Infection Disrupts the Epithelial Barrier and Activates Intrinsic Neurosecretory Reflexes in the Pig Colon. Front. Physiol..

[113] Eutamene H., Bradesi S., Larauche M., Theodorou V., Beaufrand C., Ohning G., Fioramonti J., Cohen M., Bryant A.P., Kurtz C. (2010). Guanylate Cyclase C-Mediated Antinociceptive Effects of Linaclotide in Rodent Models of Visceral Pain. Neurogastroenterol. Motil..

[114] Zhang W., Fujii N., Naren A.P. (2012). Recent Advances and New Perspectives in Targeting CFTR for Therapy of Cystic Fibrosis and Enterotoxin-Induced Secretory Diarrheas. Future Med. Chem..

[115] Devriendt B., Stuyven E., Verdonck F., Goddeeris B.M., Cox E. (2010). Enterotoxigenic *Escherichia coli* (K88) Induce Proinflammatory Responses in Porcine Intestinal Epithelial Cells. Dev. Comp. Immunol..

[116] Luo Y., Xu J., Zhang C., Jiang C., Ma Y., He H., Wu Y., Devriendt B., Cox E., Zhang H. (2019). Toll-like Receptor 5-Mediated IL-17C Expression in Intestinal Epithelial Cells Enhances Epithelial Host Defense against F4+ ETEC Infection. Vet. Res..

[117] Motyka N.I., Stewart S.R., Hollifield I.E., Kyllo T.R., Mansfield J.A., Norton E.B., Clements J.D., Bitoun J.P. (2021). Elevated Extracellular CGMP Produced after Exposure to Enterotoxigenic *Escherichia coli* Heat-Stable Toxin Induces Epithelial IL-33 Release and Alters Intestinal Immunity. Infect. Immun..

[118] Bauer D.L., Bachnak L., Limbert V.M., Horowitz R.M., Baudier R.L., D’Souza S.J., Immethun V.E., Kurtz J.R., Grant S.B., McLachlan J.B. (2023). The Adjuvant Combination of DmLT and Monophosphoryl Lipid A Activates the Canonical, Nonpyroptotic NLRP3 Inflammasome in Dendritic Cells and Significantly Interacts to Expand Antigen-Specific CD4 T Cells. J. Immunol..

[119] Hollifield I.E., Motyka N.I., Fernando K.A., Bitoun J.P. (2023). Heat-Labile Enterotoxin Decreases Macrophage Phagocytosis of Enterotoxigenic *Escherichia coli*. Microorganisms.

[120] Ma J., Hermans L., Dierick M., Van der Weken H., Cox E., Devriendt B. (2024). Enterotoxigenic *Escherichia coli* Heat Labile Enterotoxin Affects Neutrophil Effector Functions via CAMP/PKA/ERK Signaling. Gut Microbes.

[121] Loos M., Geens M., Schauvliege S., Gasthuys F., van der Meulen J., Dubreuil J.D., Goddeeris B.M., Niewold T., Cox E. (2012). Role of Heat-Stable Enterotoxins in the Induction of Early Immune Responses in Piglets after Infection with Enterotoxigenic *Escherichia coli*. PLoS ONE.

[122] Loos M., Hellemans A., Cox E. (2013). Optimization of a Small Intestinal Segment Perfusion Model for Heat-Stable Enterotoxin a Induced Secretion in Pigs. Vet. Immunol. Immunopathol..

[123] Leach S., Clements J.D., Kaim J., Lundgren A. (2012). The Adjuvant Double Mutant *Escherichia coli* Heat Labile Toxin Enhances IL-17A Production in Human T Cells Specific for Bacterial Vaccine Antigens. PLoS ONE.

[124] Sukwa N., Mubanga C., Hatyoka L.M., Chilyabanyama O.N., Chibuye M., Mundia S., Munyinda M., Kamuti E., Siyambango M., Badiozzaman S. (2023). Safety, Tolerability, and Immunogenicity of an Oral Inactivated ETEC Vaccine (ETVAX^®^) with DmLT Adjuvant in Healthy Adults and Children in Zambia: An Age Descending Randomised, Placebo-Controlled Trial. Vaccine.

[125] Bhuiyan T.R., Khanam F., Basher S.R., Dash P., Chowdhury M.I., Haque S., Harun N.B., Akter A., Karmakar P.C., Hakim A. (2025). Safety and Immunogenicity of a Recombinant Double-Mutant Heat-Labile Toxin Derived from Enterotoxigenic *Escherichia coli* in Healthy Bangladeshi Adults Delivered by Three Different Routes. Front. Bacteriol..

